# Drug-Induced Liver Toxicity and Prevention by Herbal Antioxidants: An Overview

**DOI:** 10.3389/fphys.2015.00363

**Published:** 2016-01-26

**Authors:** Divya Singh, William C. Cho, Ghanshyam Upadhyay

**Affiliations:** ^1^Department of Biology, City College of New YorkNew York, NY, USA; ^2^Department of Clinical Oncology, Queen Elizabeth HospitalKowloon, Hong Kong

**Keywords:** antioxidant, hepatoprotection, hepatotoxicity, herbal medicine

## Abstract

The liver is the center for drug and xenobiotic metabolism, which is influenced most with medication/xenobiotic-mediated toxic activity. Drug-induced hepatotoxicity is common and its actual frequency is hard to determine due to underreporting, difficulties in detection or diagnosis, and incomplete observation of exposure. The death rate is high, up to about 10% for drug-induced liver damage. Endorsed medications represented >50% of instances of intense liver failure in a study from the Acute Liver Failure Study Group of the patients admitted in 17 US healing facilities. Albeit different studies are accessible uncovering the mechanistic aspects of medication prompted hepatotoxicity, we are in the dilemma about the virtual story. The expanding prevalence and effectiveness of Ayurveda and natural products in the treatment of various disorders led the investigators to look into their potential in countering drug-induced liver toxicity. Several natural products have been reported to date to mitigate the drug-induced toxicity. The dietary nature and less adverse reactions of the natural products provide them an extra edge over other candidates of supplementary medication. In this paper, we have discussed the mechanism involved in drug-induced liver toxicity and the potential of herbal antioxidants as supplementary medication.

## Introduction

The leading cause of drug non-approval and drug withdrawal by the Food and Drug Administration (FDA) in the US is drug-induced hepatotoxicity (Ostapowicz et al., 2002; Pandit et al., 2012). More than a thousand medicines and chemicals have been reported to cause liver injury (Larrey, 2000; Biour et al., 2004; Upadhyay et al., 2010b; Porceddu et al., 2012). Drug-induced liver injury may account for approximately 10% of all cases of acute hepatitis, 5% of all hospital admissions, and 50% of all acute liver failures (Pandit et al., 2012). It is remarkable that more than 75% of cases of idiosyncratic drug reactions result in liver transplantation or death (Ostapowicz et al., 2002; Pandit et al., 2012). Drug-induced liver injury is a relatively common cause of acute liver disease and carries a mortality of around 10% (Lewis and Zimmerman, 1989; Shapiro and Lewis, 2007; Chalasani et al., 2008; Bell and Chalasani, 2009; Holt and Ju, 2010; Upadhyay et al., 2010b; Pandit et al., 2012; Björnsson et al., 2013). Inefficient drug metabolism, as is observed in the renal transplant (RT) recipients with the chronic liver disease, make them more prone to drug-induced hepatotoxicity (Contreras et al., 2007). Extensive research on drug-induced hepatic cell damage worldwide has been done in the past and is also an area of major concern at present since hepatotoxicity due to these drugs, when it becomes terminal, results in malnutrition, organ dysfunction, and death (Björnsson, 2009; Porceddu et al., 2012).

The liver plays a major role in the metabolism and removal of drugs (Pandit et al., 2012). Detoxification of drugs and xenobiotics in the liver by drug metabolizing enzymes (DMEs) is an important phenomenon in the acquisition of homeostasis (Upadhyay et al., 2007, 2008). Alteration in homeostatic status leads to a shift in the dynamic equilibrium of metabolism toward the ROS generation thereby oxidative stress leading to organ malfunction. Liver insufficiency and damage resulted from exposure to environmental toxicants, particular combinations or dosages of pharmaceuticals, and microbial metabolites are major causes of disease and death worldwide (Upadhyay et al., 2007, 2008). Intake of drugs, their metabolism and removal make a condition of dynamic equilibrium to maintain homeostasis. Phase I and phase II enzymes play a crucial role in the metabolism and detoxification of various drugs and xenobiotics. For the acquisition of dynamic homeostasis, highly tuned metabolic control of drugs or xenobiotics by xenobiotic metabolizing enzymes is needed. Any imbalance in the activity of these enzymes ultimately leads to the shifting of equilibrium toward free radical generation that could finally bind to macromolecules such as DNA to cause mutation, lipid to cause membrane damage, or proteins to alter their activities (Upadhyay et al., 2007, 2008, 2010a,b). The generation of reactive intermediates is a common event in liver damage resulting from a variety of hepatotoxic drugs and solvents (Coleman et al., 2007). The hepatotoxic reactions caused by drugs may be summarized as acute reactions (which consist of hepatocellular necrosis), cholestasis (with or without inflammation), and miscellaneous reactions; however, some drugs can cause chronic damage and may even lead to tumor growth (Table 1). In this article, we have discussed the drug-induced hepatotoxicity, factors that may add to its toxicity, mechanism, and prevention.

**Table 1:** **Common hepatotoxic reactions**.

**Drugs**	**Hepatotoxic reactions**
Acetaminophen	Acute, direct hepatocellular toxicity, chronic toxicity
Isoniazid	
Methyldopa	
Allopurinol	Miscellaneous acute reactions
Aspirin	
Quinidine	
Sulfonamides	
Valproate	
Amiodarone,	Chronic toxicity
Methotrexate	
Niacin	
Rifampicin	
Pyrogallol	
Vitamin A	
Chlorpropamide Erythromycin-estolate Phenylbutazone	Acute cholestasis, phenothiazine type
Diclofenac	Acute, idiosyncratic hepatocellular toxicity
Halothane-related anesthetics Indomethacin	
Phenytoin	
Propylthiouracil	
Hydrocarbons	Acute, direct hepatocellular toxicity
Tratcycline	
Methyltestisterone	Acute cholestasis, steroid type
Oral contraceptives	

## Factors affecting drug-induced hepatotoxicity

**Figure 1:**
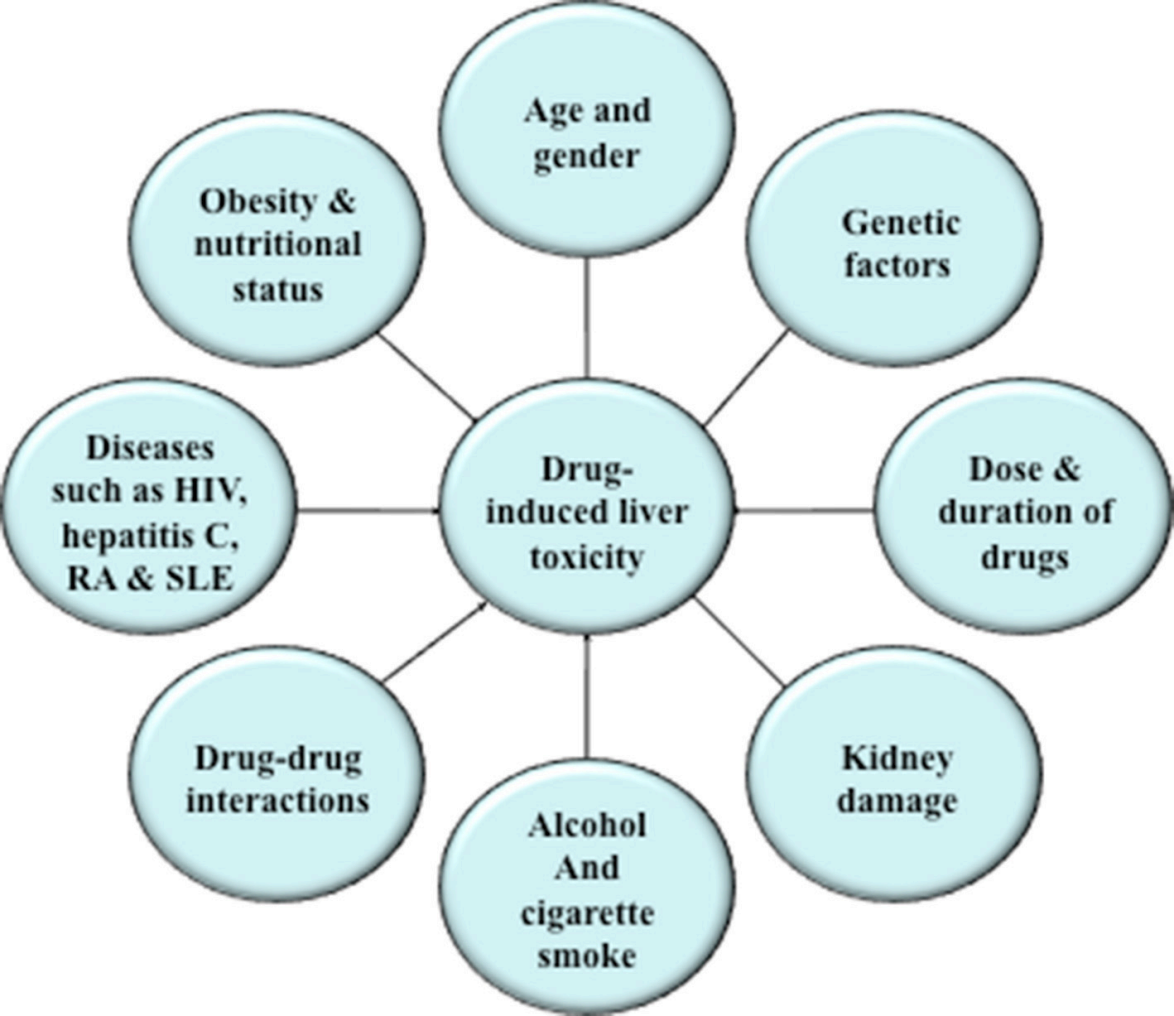
**Factors affecting drug-induced hepatic toxicity**. Various factors, such as, advancing age, gender, lifestyle factors, obesity, nutritional status, genetic background, dose, and duration of drugs may increase the risk of drug-mediated hepatotoxic reactions.

There are various factors that enhance a person's susceptibility to a potentially hepatotoxic drug (Figure 1). Advancing age, gender, lifestyle factors, obesity, nutritional status, genetic background, dose, and duration of drugs may affect the risk of drug-mediated hepatotoxic reactions. Persons suffering from other diseases, such as, human immunodeficiency virus, hepatitis C, rheumatoid arthritis, and systemic lupus erythematosus, are more prone to toxic drug reactions. Drug composition and drug-drug interaction may also be a reason for increased risk of drug-induced hepatotoxicity. For example, certain drugs containing a nitro-aromatic moiety or drugs interacting with nuclear receptors such as phenobarbital may cause organ-selective toxicity or may potentiate the toxicity of other drugs (Yamazaki et al., 2005; Boelsterli et al., 2006).

## Drug-induced damage

A rising number of cases of acute liver failures are reported every year in the United States, and drugs contribute majority of them, e.g., acetaminophen and idiosyncratic reactions due to other medications (Pandit et al., 2012). Two to five percent of jaundice cases and over 10% cases of acute hepatitis are also contributed by drug-mediated side reactions (Pandit et al., 2012). Several drugs and chemicals have been withdrawn from the market worldwide due to their hepatotoxic reactions (Bakke et al., 1995; Shah, 1999; Lee, 2003; Mohapatra et al., 2005; Upadhyay et al., 2010a,b; Pandit et al., 2012), yet epidemiological studies are alarming and suggest that the drug trial process should be more rigorous. Some drugs have been extensively studied in humans and animals to elucidate the biochemical and molecular mechanism of drug-induced hepatotoxicity (Upadhyay et al., 2007, 2008, 2010a,b). Most of the drugs have a signature effect, and exhibit a pattern of liver injury. Nevertheless, certain drugs like rifampicin may produce all kind of symptoms associated with liver injury, ranging from cholestasis, hepatocellular injury, to even isolated hyper-bilirubinemia. Therefore, the knowledge of the most commonly used drugs/agents, such as, anti-tubercular drugs, acetaminophen, diclophenac, pyrogallol, statins and so forth, and a high index of suspicion are essential in diagnosis.

## Rifampicin

Hepatitis has been reported to occur 0.46% of individuals undergoing anti-tubercular therapy and receiving anti-tubercular drugs. The rate of hepatotoxic reaction was much higher in Indian patients as compared to that reported from developed countries (Ramachandran, 1980; Alexander et al., 1982; Mindie and Gabriel, 2002). It is readily absorbed from the gastrointestinal tract (90%) and most of it is bound to plasma proteins in circulation. The involvement of oxidative stress in rifampicin-induced hepatotoxicity has been demonstrated previously in experimental rats (Sodhi et al., 1998). Rifampicin is a potent inducer of cytochrome P450 action and enhances the covalent binding of reactive metabolites of acetyl hydrazine to the macromolecules of hepatocytes leading to hepatic cell damage (Powell-Jackson et al., 1982; Sinha, 1987). Additionally, desacetylrifampicin, another reactive metabolite of rifampicin, also contributes to some of its adverse effects. It has been reported to modulate the membrane permeability and cause membrane damage (Rana et al., 2006). Its prolonged exposure significantly decreases glucose-6-phosphatase activity, which could be a reason for the increased level of lipid peroxidation (Koster and Slee, 1980; Saraswathy and Shyamala Devi, 2001). Anti-tubercular drugs increase intracellular calcium concentration leading to the induction of phospholipase A2 which degrade membrane phospholipids (Karthikeyan, 2005; Tasduq et al., 2005). Additionally, CYP2E1 activation and fatty acid accumulation in the liver either due to excessive supply of lipids to the liver or interference with lipid deposition, has been documented in the anti-tubercular drug-induced liver disorders (Anundi et al., 1993; Farombi et al., 1999; Upadhyay et al., 2007). Anti-tubercular drugs also induce hypercholesterolemia that might be due to increased uptake of LDL from the blood, by the tissues (Kissler et al., 2005; Santhosh et al., 2006).

## Isoniazid

Isoniazid, an anti-tubercular drug, is used alone or in combination with other drugs to eliminate the active (growing) bacteria. Usually, the therapy with isoniazid is continued for a longer time (6–12 months) since the bacteria may exist in a resting state for a longer period of time. The studies have indicated severe and fatal hepatitis with isoniazid therapy (Huang et al., 2003; Saukkonen et al., 2006). The frequency of hepatotoxic reactions are higher in aged patients over 65 years. Additionally, daily alcohol consumption increases the risk of hepatitis. In the patients receiving isoniazid, the symptoms of hepatic damage appear late, usually after 3 months of treatment.

In the liver, isoniazid is metabolized primarily by N-acetyl transferase 2 (NAT-2) to acetyl-isoniazid, which subsequently is converted to mono-acetyl hydrazine (MAH) and non-toxic diacetyl hydrazine, as well as other minor metabolites (Huang et al., 2002; Saukkonen et al., 2006). Studies have revealed that reactive metabolites of MAH are toxic to tissues due to ROS generation (Mitchell et al., 1975; Saukkonen et al., 2006). Isoniazid inhibits glutathione biosynthesis, activities of antioxidant glutathione peroxidase and catalase activity in rats (Sodhi et al., 1996; Attri et al., 2001; Saukkonen et al., 2006). Furthermore, acetyl-hydrazine, a metabolite of isoniazid, causes damage to hepatic cells by covalently binding to liver macromolecules (Mitchell et al., 1975; Saukkonen et al., 2006). In an epidemiological study, it has been reported that homozygous CYP2E1 c1/c1 host gene polymorphism, which results in enhanced CYP2E1 activity, causes higher risk of hepatotoxicity in patients (Huang et al., 2003; Saukkonen et al., 2006).

## Acetaminophen

Acetaminophen (APAP) is a commonly used analgesic and antipyretic, which is relatively safe at recommended therapeutic doses (Blazer and Wu, 2009). However, its associated hepatotoxicity is a major concern and the leading cause of drug-induced liver failure in many countries when used at high doses (Larson et al., 2005; Mitka, 2014; Wang et al., 2015). Acetaminophen has been extensively studied in order to understand the mechanism of drug-induced hepatotoxicity. Its hepatotoxicity pattern is different in various age groups for example the hepatotoxic incidences are less in neonates than in older children and adults. It has been suggested that in neonates, the oxidative enzyme activity is limited therefore the incidences are less common (Jacqz-Aigrain and Anderson, 2006). The acetaminophen-induced liver injury leads to a functional suppression of the immune system as dictated by the hindrance of a deferred hypersensitivity reaction to dinitrochlorobenzene (Masson et al., 2007). Subsequent studies with adrenalectomized mice, recommended a role of corticosterone in the exhaustion of lymphocytes taking after APAP-instigated liver damage (Masson et al., 2007). Acetaminophen is metabolized to N-acetyl-p-benzoquinone imine by CYP enzymes, which is subsequently detoxified by reduced glutathione (GSH) to a threshold concentration, after which GSH depletion occur leading to the covalent binding of the metabolite to the macromolecules (Reid et al., 2005; Wolf et al., 2007; Olaleye and Rocha, 2008; Saito et al., 2010; McGill et al., 2012). APAP overdose has been reported to induce massive necrosis in the liver in animal models. It is remarkable that only those hepatocytes undergo necrosis in which acetaminophen-protein adducts formation occur (Hinson et al., 2004; Saito et al., 2010). It is also suggested that the APAP toxicity propagates through nitric oxide (NO), which scavenges superoxide to produce peroxynitrite, thereby causing protein nitration (3-ntrotyrosine) and tissue injury (Jaeschke et al., 2002; Hinson et al., 2004). Three-Nitrotyrosine is usually detoxified by conjugation with reduced GSH. Thus, acetaminophen toxicity occurs with increased oxygen/nitrogen stress (Hinson et al., 2004).

## Pyrogallol

Pyrogallol is an anti-psoriatic drug and has been reported to cause liver damage in mouse and rat models (Gupta et al., 2002; Upadhyay et al., 2007, 2008, 2010a,b). In the presence of metal ions under certain conditions *in vitro*, pyrogallol causes oxidative damage to macromolecules (Singh et al., 1994). Pro-oxidant action of pyrogallol is suggested to be the major cause of harmful effects such as mutagenesis, carcinogenesis and hepatotoxicity (Akagawa et al., 2003; Upadhyay et al., 2007). It is suggested that the auto-oxidation property of pyrogallol due to the attack by reactive oxygen species such as •OH, ${\text{O}}_{2}^{-}$, and hydrogen peroxides contribute greatly to its pro-oxidant actions (Cao et al., 1997; Hayakawa et al., 1997; Mochizuki et al., 2002; Akagawa et al., 2003). Studies have now established the involvement of toxicant responsive genes for example CYP1A2, CYP2E1, glutathione-*S*-transferase, glutathione reductase, and glutathione peroxidase in pyrogallol-induced membrane damage and hepatotoxicity (Upadhyay et al., 2007, 2008, 2010a,b).

## Diclofenac

Diclofenac-mediated liver toxicity is a prime example of idiosyncratic liver injury (Mitchell et al., 1973) and the increased hepatotoxicity markers have been noted in about 15% of patients receiving diclofenac regularly. Elderly females are more susceptible to diclofenac-induced liver injury. (Banks et al., 1995; Kaplowitz, 2001; Mitchell and Hilmer, 2010) Diclofenac predominantly exhibits hepatocellular pattern of liver injury; however, cholestatic pattern of liver injury and cases resembling autoimmune hepatitis have also been observed (Aithal, 2004). Metabolism of diclofenac by Cyp2C8/9 or by UDP-glucuronosyltransferase-2B7 results in unstable intermediate compounds that modify proteins covalently and increase the risk of hepatotoxicity. Additionally, covalent binding of reactive metabolites to “self” proteins results in the formation of neoantigens, which could be recognized by helper T cells leading to their activation and an effector-cell response. It is remarkable that hepatocytes express MHC I molecules on their surfaces and may present diclofenac adducts, making them prone for T-cell mediated liver injury. Alternatively, diclofenac adducts on the plasma membrane of hepatocytes may be recognized by B cells resulting in their maturation into plasmacytes, the secretion of antibodies and ultimately immunological destruction of hepatocytes (Aithal, 2011).

## Statins/HMG-CoA reductase inhibitors

Statins are the widely used drugs in the Western countries including United States. Statins inhibit the process of cholesterol biosynthesis by competitively inhibiting HMG-CoA reductase. Further, they also decrease the low-density lipoprotein levels and increase the atherosclerotic plaques stability (Jacobson, 2006). The use of higher statin doses is shown to exhibit biochemical abnormalities of liver function as indicated by moderate elevations of hepatotoxicity markers in animal models (Horsmans et al., 1990). High doses of derivatives of statins, lovastatin and simvastatin, have been shown to cause significant hepatocellular necrosis in rabbits and guinea pigs respectively (Horsmans et al., 1990). Nevetheless, hepatocellular necrosis by statins is rarely observed in humans (Alonso et al., 1999).

## Other drugs

There are some other commonly used drugs that cause liver damage, for example, valproic acid, an antiepileptic drug, may cause liver damage in approximately 20% patients; antibiotics namely ciprofloxacin, erythromycin, amoxicillin may produce symptoms ranging from jaundice, acute hepatotoxicity to liver malfunction; chlorpromazine is found to produce symptoms of jaundice; amiodarone causes acute hepatotoxicity in both animal and human studies; oral contraceptives may cause jaundice and cholestatic liver injury and so forth.

## Understanding the mechanism of hepatotoxicity

**Figure 2:**
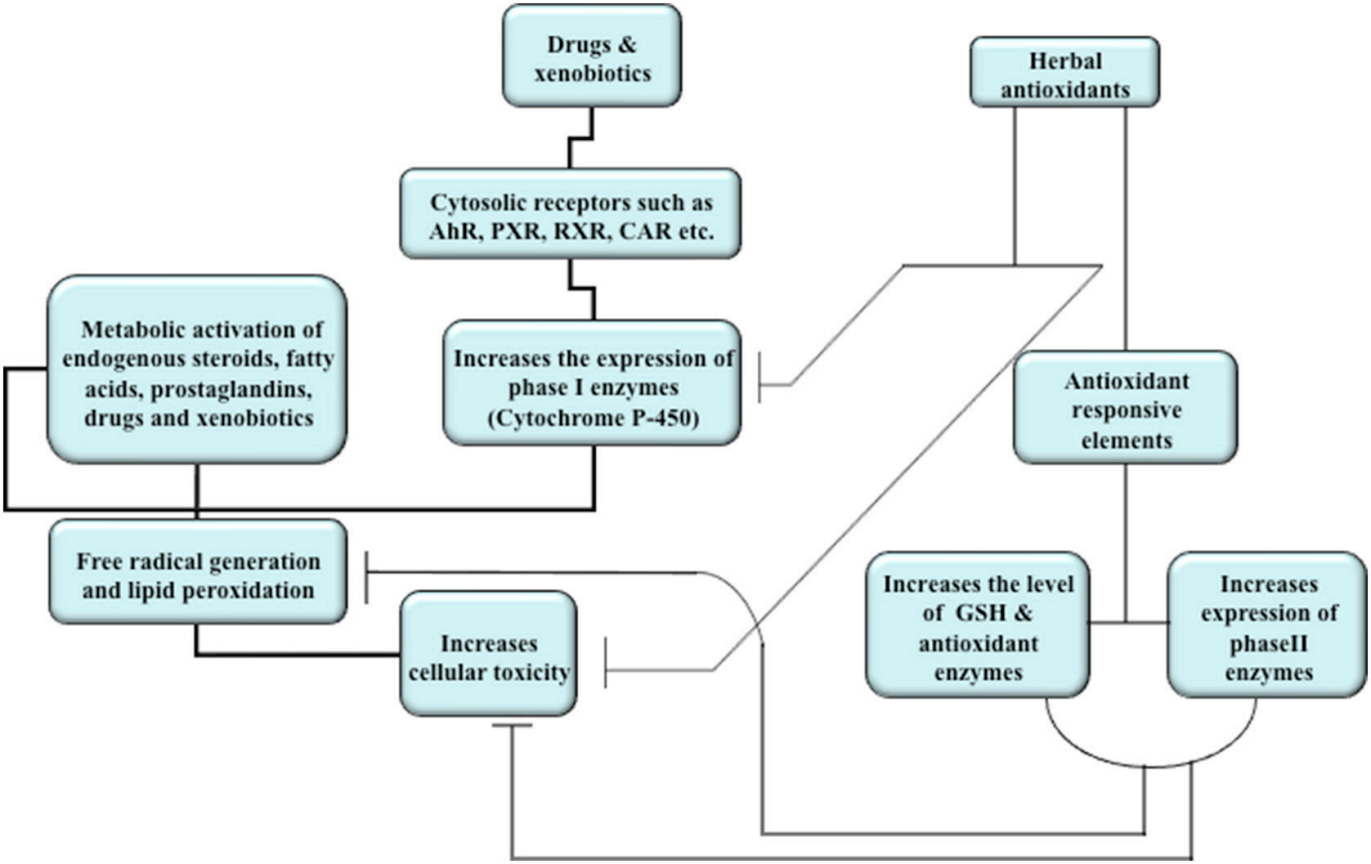
**Overview of mechanism of drug and xenobiotic metabolism and effects of herbal antioxidants**. Induction of nuclear receptors by drugs and xenobiotics lead to translocation in the nucleus where they increase the expression of cytochrome P450 enzymes. The activity of these enzymes generates reactive metabolites and free radicals which in turn bind to macromolecules, cause membrane lipid peroxidation, and increase cellular toxicity. Natural products increase the expression of phase II enzymes, the level of intracellular antioxidant (GSH), and antioxidant enzymes. The natural products may also inhibit the activities of cytochrome P450 enzymes in order to attain dynamic homeostasis and reduce cellular toxicity.

Mechanism of drug-induced hepatotoxicity is variable and complex (Figure 2). Some drugs are directly toxic and begin to exhibit hepatotoxic reactions, which are dose related, within hours of exposure, whereas others may produce liver injury only in susceptible people and symptoms appear after few days or weeks. These reactions are rarely allergic or more accurately described as idiosyncratic. Drug-drug interaction, though the drugs are not hepatotoxic themselves, may also play a critical role in the propagation of toxicity (Yamazaki et al., 2005). The pathogenesis of drug-induced toxicity mediated by reactive metabolites has been a center of research enthusiasm since spearheading examinations in the 1950s uncovered the connection between these metabolites and chemical carcinogenesis (Park et al., 2005). A major cause of hepatotoxic reactions may be drug-induced intrahepatic cholestasis, which often occurs during the drug discovery and development process. The vital roles of ROS in the cellular damage are widely investigated and it has been suggested that the covalent binding of ROS as well as reactive intermediates to macromolecules could likely contribute to the severe harmful drug reactions (Racknagel et al., 1989; Masubuchi et al., 2007). There are several studies that suggest the generation of reactive metabolites and free radicals from hepatotoxic drugs (Racknagel et al., 1989; Park et al., 2005). Membrane lipid peroxidation is directly related to the depletion of tissue GSH (an intracellular antioxidant) leading to the altered functional integrity of these structures and if the damage is sever, it could be fatal (Ross, 1988; Guven and Gulmez, 2003). Membrane lipid peroxidation may lead to alteration in membrane fluidity and permeability, enhanced rates of protein degradation, and ultimately cell death (García et al., 1997). The assumption is upheld by the way that oxidative damage to erythrocytes causes loss of membrane capacity by enhancing lipid peroxidation (LPO) and modifying the erythrocyte antioxidant framework (Vajdovich et al., 1995). The concentration of intracellular GSH, therefore, is the key determinant of membrane integrity and the extent of toxicant-induced hepatic cell injury (Ross, 1988; Guven and Gulmez, 2003).

## Drug-metabolizing enzymes (DMEs)

DMEs and transporters are key factors that affect the disposition of xenobiotics (Cheng et al., 2005). These DMEs include mix function oxidases and phase II backup machinery that are actively involved in the detoxification process. Recently, the roles of transporters (Phase III enzymes) also have been implicated in the detoxification procedure.

## Phase I enzymes (mixed function oxidases)

Cytochrome P450 (CYP), a super gene family of heme-containing, mixed function oxidase enzymes, are involved in the metabolism of endogenous steroid hormones, vitamins, endobiotics (fatty acid derivatives), various drugs/xenobiotics, and are readily inducible by many exogenous and endogenous substances (Nelson et al., 1996; Ahmed and Pawar, 1997). The CYP-catalyzed oxidation of xenobiotics generate a highly electrophilic intermediates capable of forming covalent adducts with critical cell macromolecules, such as thiol-containing membrane proteins that direct Ca^++^ homeostasis (Bellomo and Orrenius, 1985; Dahm and Jones, 1996; Vessey, 1996). The activation of increased intracellular calcium may be the regular pathway promoting cell death. CYP-mediated reduction of halogenated hydrocarbons, for example, carbon tetrachloride or halothane, can likewise create free radical intermediates, which can straightforwardly harm cell layers through lipid peroxidation, or can target nucleophilic DNA deposits (Thor and Orrenius, 1980; De Groot and Noll, 1983; Racknagel et al., 1989; Dahm and Jones, 1996).

Phase I reactions, catalyzed by various drugs and xenobiotics, are mediated by various isoforms of CYPs and are regulated through various nuclear receptors. Phase I enzyme impelling by classes of microsomal inducers happens by means of enactment of transcription factors, for example, aryl hydrocarbon receptor (AhR), constitutive androstane receptor (CAR), pregnane X receptor (PXR) and peroxisome proliferator-actuated receptor α (PPARα) (Cheng et al., 2005). Various isoforms of CYP are reported to be involved in the metabolism and therefore bioactivation of drugs. CYP3A, CYP2D, and CYP2C sub-families are involved in the metabolic activation of 50%, 25%, and 20%, respectively, of the currently used clinical drugs (Rendic, 2002; Deng et al., 2008). CYP1A2, 2C7, 2C11, 2D2, 2E1, 2B1/2, and 3A1/2 are the major CYP isoforms involved in the drug metabolism in rats (Kobayashi et al., 2002; Deng et al., 2008). Involvement of CYP2E1 and 1A2 in the acetaminophen and pyrogallol activation has been well established in animal models (Raucy et al., 1989; Snawder et al., 1994; Upadhyay et al., 2007). The activation of paracetamol to N-acetyl-para-benzoquinoneimine (NAPQI) is catalyzed by CYP2E1, 3A4, and 1A2 in humans (Raucy et al., 1989; Snawder et al., 1994). The involvement of CYP2E1 in rifampicin and ethanol-induced liver toxicity has also been reported (Jaeschke et al., 2002; Upadhyay et al., 2007).

## Regulation of phase I enzymes: nuclear receptors

Regulation of phase I enzymes, particularly CYPs is coupled with various transcription factors also called nuclear receptors (NR). These are the largest known family of transcription factors that modulates the tissue gene expression and are associated largely with metabolism, developmental function, and cell differentiation. There are 49 known members of the nuclear receptor superfamily and each share key structural features (Urquhart et al., 2007; Woods et al., 2007). These are the targets of approximately 10–15% pharmaceuticals and explore about $400 billion global pharmaceutical markets (Woods et al., 2007). The majority of the nuclear receptors target on various proteins included in xenobiotic metabolism, in particular, CYP enzymes (Waxman, 1999; Honkakoski and Negishi, 2000; Johnson and Klaassen, 2002). Several receptors require heterodimerization with retinoid X receptor (RXR) (Mangelsdorf and Evans, 1995). Activation of nuclear receptors, for example, aryl hydrocarbon receptors (AhR), RXR, pregane X receptors (PXR), and peroxisome proliferator-initiated receptors (PPAR) are crucial for some clinically imperative drug-drug interactions. Activation of phase I enzymes by agonists can be ascribed for activation of signal transduction pathways. Various microsomal protein inducers display their effects through prompting of diverse orphan nuclear receptors, therefore enhancing target gene expression (Denison and Nagy, 2003; Sonoda et al., 2003). For example, 2,3,7,8-tetrachlorodibenzo-p-dioxin (TCDD) binds to the AhR, which releases the AhR from the cytosolic tethering protein HSP90, permitting AhR translocation to the nucleus, heterodimerization with aryl hydrocarbon receptor nuclear translocator (ARNT), and the binding to xenobiotic response elements on the CYP1A1 promoter. Also, CAR ligands affect CYP2B10, PXR ligands incite CYP3A11, and peroxisome proliferator-initiated receptor α (PPAR α) ligands activate CYP4A14 (Cheng et al., 2005). The pregnane X receptor (PXR, NR1I2), a member of the nuclear receptor superfamily, is activated by a lot of compounds and natural products. It is involved in the regulation of various CYP isoforms such as CYP 3A4, which is involved in the metabolism of about 60% of all prescription drugs (Lehmann et al., 1998; Guengerich, 1999; Kliewer, 2003). PXR sometimes mediates the protective effects also in case of some herbal remedies besides its undesirable effects in patients on combination therapy (Staudinger et al., 2006).

## Phase II enzymes

Detoxification by phase II enzymes eliminates the reactive intermediates from cellular environments and therefore decreases the burden of bio-molecular adducts on cellular homeostasis (Habig et al., 1974; Moon et al., 2006). Phase II enzymes are mainly involved in the conjugation of activated pro-toxicants with endogenous bio-molecules like GSH or glucuronic acid which reduces toxicity and increases water solubility (Habig et al., 1974; Moon et al., 2006). Glutathione-*S*-transferases (GST), a crucial Phase II enzyme, initiates the detoxification process by catalyzing the conjugation of –SH moiety of glutathione to xenobiotics, thereby neutralizing the electrophilic sites and rendering the products more water-soluble. Glutathione conjugates are thought to be metabolized further by cleavage of the glutamate and glycine residues, followed by acetylation of resultant free amino groups of the cysteinyl residue, to produce the final product, a mercapturic acid (Boyland and Nery, 1969; Wood and Woodcock, 1970).

## Regulation of phase II enzymes

Phase II enzymes are regulated by regulatory elements called ARE (anti-oxidant response elements), which are situated upstream to promoter regions (Hayes and McMahon, 2001). Regulation is coupled with the activation of ARE through activation of a transcription factor, nuclear erythroid factor 2—related factor 2 (Nrf2; Lee and Surh, 2005). Nrf2 either upregulates or inhibits transcription of phase II enzymes through ARE by the heterodimerization with an array of leucine b-zip family members such as small maf proteins, fos, jun, and so forth (Jaiswal, 2000; Cheng et al., 2005). Nrf2 is tightly coupled with Keap1, which acts as a negative regulator of Nrf2 and as a sensor of xenobiotic and oxidative stresses (Motohashi and Yamamoto, 2004). Nrf2 activation leads to its release from Keap1 followed by translocation to the nucleus, heterodimerization to other leucine zipper proteins, and binding to ARE in order to transcriptionally activate the downstream targets (Motohashi and Yamamoto, 2004). Nrf2 activation is triggered by the ROS modulation in cells via the interaction of a various signaling molecules specified by cell types (Hayes and McMahon, 2001). Therefore, most chemopreventive operators are the modulators of cell ROS and subsequently activate Nrf2 pathway, which thusly incite phase II detoxifying response (Hayes and McMahon, 2001). In addition to the dynamic equilibrium of Phase I and Phase II enzymes, reduced GSH, glutathione peroxidase (GPx) and catalase (CAT) are also critical in acquisition of normal cellular physiology as these are involved in the elimination of lipid peroxides and toxic oxygen radicals formed during the cellular metabolism (Trackshel and Maines, 1988; Tanaka et al., 2004; Pal et al., 2006).

Another adoptive cellular response against drugs/toxicants is the induction of heme oxygenase-1 (HO-1), the rate-limiting enzyme in the breakdown of heme into carbon monoxide (CO), iron and bilirubin (Farombi and Surh, 2006). HO-1 is instigated by oxidative stress stimuli and its enactment is considered an adoptive survival response (Immenschuh and Ramadori, 2000). HO-1 impelling possibly provides security against oxidative damage, for example, in liver ischemia/reperfusion secondary to transplantation or hemorrhage/resuscitation. Induction of HO-1 controls the intracellular levels of “free” heme (a prooxidant), increases the delivery of biliverdin (an antioxidant), enhances nutritive perfusion, and cultivates the amalgamation of the Fe-binding protein ferritin to ensure the protection of cells against oxidative stress (Bauer and Bauer, 2002). CO and biliverdin/bilirubin, which are generated as a product of HO-1 activity, have been accounted for the protective effects in a few organs, including the liver (Farombi and Surh, 2006). Additionally, HO-1 is an intense defensive component for cytokine-and CD95-intervened apoptotic liver damages (Sass et al., 2003). Nevertheless, the heme oxygenase gives a defensive shield against oxidative stress to a certain level of expression and a large amounts of HO-1 may even sensitize the cells to oxidative damage by increasing the level of “free” reactive iron (Bauer and Bauer, 2002).

## Role of transporters

The reduction in expression of uptake transporters, and augmentation in expression of export transporters, as well as detoxification enzymes, may prevent the hepatocytes from accumulating potentially toxic products (Aleksunes et al., 2005). Therefore, the instances and propagation of liver injury appears to be a coordinated phenomenon involving both transport and detoxification genes (Aleksunes et al., 2005). The phenomenon of extraction and subsequent excretion of drugs and their toxic metabolites from portal blood is performed by basolateral and canalicular transporters in hepatocyte plasma membranes (Arrese and Accatino, 2002). Uptake carriers, such as organic anion-transporting polypeptides (Oatps) and the sodium/taurocholate-cotransporting polypeptide (Ntcp), and export transporters such as multidrug resistance proteins (Mdrs), multidrug resistance-associated proteins (Mrps), bile salt export pump (Bsep), and breast cancer resistance protein (Bcrp) are actively involved in the process of transportation of drugs/xenobiotics from absorption till excretion (Arrese and Accatino, 2002; Geier et al., 2002; Song et al., 2003). The transporters perform specialized functions, for example, canalicular transporters (Mrp2, Mdrs, Bcrp, and Bsep), are involved in the excretion of xenobiotics and their metabolites from hepatocytes into bile, and basolateral transporters (Mrp 1, 3–6) mediate the efflux of drugs and chemicals from hepatocytes into blood (Arrese and Accatino, 2002; Geier et al., 2002; Song et al., 2003). Various reports are available online implicating the involvement of these transporters in the xenobiotics metabolism and propagation of cell injury (Arrese and Accatino, 2002; Geier et al., 2002; Song et al., 2003; Ghanem et al., 2004; Heijne et al., 2004).

## Herbal medication

Herbal antioxidants have attracted the researchers due to its potential and efficacy against drug-induced liver toxicity. Natural products used in China and India as traditional medicine for the treatment of liver disorders are of great interest in these days. These are the potential sources for new therapeutic agents that could be used in the prevention of hepatic injuries. Natural products rich in triterpenes, flavonoids or polyphenols, have been now established as powerful hepatoprotective agents in experimental liver-injury cell and animal models (Table 2; Gupta et al., 2002, 2004; King and Cousins, 2006; Upadhyay et al., 2007, 2008, 2010a,b). The basis behind the protection provided by the natural products is hypothesized to be their antioxidant property through which they remove the free radicals from the cellular environment and therefore provide protection against ROS mediated damage to membrane lipids and macromolecules (Figure 2; Gupta et al., 2002, 2004; Upadhyay et al., 2007, 2008, 2010a,b). Additionally, the protective potential of natural products is also contributed by its interaction with various CYP isoforms, its capability to increase GSH biosynthesis, level of Phase II/antioxidant enzymes and to inhibit the entry of toxins to the cells (Figure 2; Gupta et al., 2002, 2004; King and Cousins, 2006; Upadhyay et al., 2007, 2008, 2010a,b; Yarnell and Abascal, 2007). Some of the natural products containing polyphenols are considered as potential chemopreventive and hepatoprotective agents. In the last decade, several studies have been conducted to investigate the mechanism of action of natural products at biochemical, genomic and proteomic levels. The most extensively investigated natural products for hepatoprotection are silymarin, resveratrol, curcumin and gingko due to their high efficacies, low or no toxicity and easy availabilities.

**Table 2:** **Natural products, their active ingredients and effects**.

**Natural products**	**Major constituents**	**Effect**	**References**
Barley grass	2-0-glycosylisovitexin (2-0-GIV), flavone-C-glycosides, saponarin and lutonarin contain natural SOD	Protect damage by inhibition of fat oxidation (lipid peroxidation) and by increasing antioxidant capacity	Duarte, 1995; Markham and Mitchell, 2003
Carrot	Xanthophyll, betacarotene, and other antioxidant carotenoids	Protects against damage due to powerful antioxidant capacity of xanthophyll, betacarotene	Duarte, 1995
Citrus fruit	Flavonoids and vitamin C	Provides protection against damage by its antioxidant capacity	King and Cousins, 2006
Eleuthero root (also known as Siberian ginseng)	Four shogaols contain antioxidants	Protects against damage due to its antioxidant and anti-lipid peroxidative activities	Bol'shakova et al., 1997; DerMarderosian, 2001; Yu et al., 2003
Ginger root	About 40 different bioflavonoids, including proanthocyanidins and quercetin	Protects against damage due to its antioxidant and anti-lipid peroxidative activities	DerMarderosian, 2001; Halvorsen et al., 2002; Kim et al., 2002
Ginkgo leaf	About 40 different bioflavonoids, including proanthocyanidins and quercetin	Protects against damage due to its antioxidant and anti-lipid peroxidative activities; also inhibits nuclear factor-kappa B and activator protein 1 activation, and adhesion molecule expression in HAECs therefore effective against atherosclerosis disease	DerMarderosian, 2001; Chen et al., 2003; DeFeudis et al., 2003; King and Cousins, 2006
Grape seed/skin	A variety of antioxidant substances and is over 90% proanthocyanidins	Protects against damage by blocking free radical generation and lipid peroxidatin as well as by regulating bcl-X(L) gene, DNA damage and presumably by reducing oxidative stress	Bagchi et al., 2000; Young et al., 2000; DerMarderosian, 2001
Kudzu root	Isoflavone known as puerarin (its crude form is more efficient than puerarin)	Protects against damage due to its antioxidant capacity	Guerra et al., 2000; Allen et al., 2003; Rezvani et al., 2003
Milk thistle seed	Silymarin (major constituents silybinin)	Protects against damage due to its antilipid peroxidative capacity and antioxidant nature. It also inhibits CYP isoforms and increases antioxidant enzymes	Ben-Amotz et al., 1998; Shimizu, 2001; Kvasnicka et al., 2003; Upadhyay et al., 2007
Rosemary leaf	Flavonoids such as cirismarin, diosmin, hesperidin, homoplantiginin, and phegopolin	Protects against damage due to its antioxidant capacity and have analgesic property for myalgias and neuralgias	Duarte, 1995; Gruenwald et al., 2000
Schisandra fruit	At least 9 dibenzocyclooctene lignans	Protects against damage due to its antioxidant and anti-lipid peroxidative activities and decrease the extent of membrane fluidity of liver microsomes	Lu and Liu, 1992
Tomato	Lycopene and vitamin C	Protects against damage due to its antioxidant capacity	King and Cousins, 2006
Turmeric root	Curcuminoids	Protects against damage due to its antioxidant capacity and also inhibits different molecules involved in inflammation such as phospholipase, lipooxygenase, cyclooxygenase 2, leukotrienes, thromboxane, prostaglandins, nitric oxide, collagenase, elastase, hyaluronidase, monocyte chemoattractant protein-1, interferon-inducible protein, tumor necrosis factor, and interleukin-12	DerMarderosian, 2001

## Silymarin

Silymarin, isolated from the seeds of milk thistle (Silybum marianum), is an unique flavonoid-complex containing silybinin, silydianin, and silychristin and have been extensively studied (Flora et al., 1996; Madrigal-Santillán et al., 2013, 2014). Silibinin is major constituent of silymarin. Two main mechanisms of action of silymarin have been proposed based on its cell-regenerating function and cytoprotective effect. Cytoprotection is mediated by its antioxidant properties and direct interaction with cell membrane components (Muriel and Mourelle, 1990; Mira et al., 1994). Inhibition of lipid peroxidation, as reported in various *in vitro* studies using erythrocytes (Valenzuela et al., 1987), isolated and cultured hepatocytes (Joyeux et al., 1990; Miguez et al., 1994), and human mesangial cells, is accepted as one of silymarins major protective mechanisms. Silymarin has been also reported to have anti-inflammatory (De la Puerta et al., 1996), anti-fibrotic (Fuchs et al., 1997), and anti-proliferative effects (Scambia et al., 1996). Diverse biochemical reactions, such as, the incitement of the synthetic rate of ribosomal RNA (rRNA) species through the induction of polymerase I and rRNA transcription, shielding the cell from free radical-mediated injury and blockage of the uptake of toxins, also contribute to the protective potential of silymarin (Sonnenbichler and Zetl, 1986; Sonnenbichler et al., 1999; Wellington and Jarvis, 2001). Silymarin offers protection against enlarged liver by inhibiting 5-lipoxygenase, production of leukotrienes and generation of free radicals in Kupffer cells. Moreover, silybin, a major component of silymarin, protects against the membrane lipid peroxidation and cellular damage in the mouse hepatocytes (Yin et al., 2011; Bahmani et al., 2015).

Silymarin has been shown to be effective in combating against drug-induced hepatotoxicity in various animal models (Gupta et al., 2002, 2004; Upadhyay et al., 2007). Hepatoprotective potential of silymarin is suggested to be due to its antioxidant, cell regeneration and cytoprotection activity (Sonnenbichler et al., 1999; Gupta et al., 2002, 2004; Upadhyay et al., 2007; Ahmad et al., 2013; Yang et al., 2014b). It is equally efficient in animals as well as in humans. It increases the level of glutathione and glutathione peroxidase in the serum of patients and animals. Although silymarin has low oral absorption, oral dosages of 420 mg/day have shown some therapeutic potential, with good tolerability in alcoholic cirrhosis patients. Moreover, daily dose of silybin (20–48 mg/kg) is supposed to be an antidote for acute mushroom poisoning by Amanita phalloides. Some isoforms of Silybin have been shown to possess the strongest anti-NF-κB and anti-HCV activity (Polyak et al., 2007). It has been shown to offer protection against prostate cancer by the inactivation of erbB1-SHC (Src homology 2 domain containing) signaling pathway and induction of CDKIs, and a resultant G1 arrest (Zi et al., 1998; Zi and Agarwal, 1999). Additionally, it inhibits xanthine oxidase and mRNA expression of TNF-α, which plays critical role in mouse skin tumorigenesis (Zi et al., 1997; Sheu et al., 1998). At lower non-toxic concentrations, it inhibits transformation in cultured rat tracheal epithelial cells treated with benzo[a]pyrene, by which chemopreventive compounds that act at early stages of the carcinogenic process, could be identified (Steele et al., 1990; Kohno et al., 2002). Protective potential of silymarin against rifampicin and pyrogallol-induced hepatotoxicity has been found to be due to its modulatory effect on the augmented level of CYPs and attenuated level of phase II and antioxidant enzymes (Upadhyay et al., 2007). In a clinical trial with over 2000 patients with chronic liver diseases, administration of silymarin extract for 8 weeks resulted in a significant decrease in liver damage index in approximately 88% of the patients (Nasri et al., 2013). It was remarkable that some minor side effects were observed only in less than 1% of patients (Nasri et al., 2013).

## Resveratrol

Resveratrol (trans-3,4′,5-trihydroxy-trans-stilbene) is a natural polyphenol present in significant amounts in peanuts, the skin of grapes and red wine. Potential health benefits of resveratrol have been widely investigated in past few years. Its activity in chemoprevention, cardiovascular diseases and neurodegenerative disorders has been reported in preclinical studies (Surh, 1999; Frémont, 2000; Ignatowicz and Baer-Dubowska, 2001; Latruffe et al., 2002; Gescher and Steward, 2003; Srivastava et al., 2013). Mechanism based inactivation of CYP2E1 and CYP1A2 by resveratrol has been reported previously (Chang et al., 2001; Mikstacka et al., 2002; Upadhyay et al., 2008). Further, it has been demonstrated that the metabolic hydroxylation of resveratrol by CYP1B1, results in its conversion to piceatannol, a tyrosine kinase inhibitor and a compound of known anticancer activity (Geahlen and McLaughlin, 1989; Mikstacka et al., 2002; Potter et al., 2002). The substitution of the hydroxy with methoxy groups in resveratrol molecules may increase its lipophilicity and binding to the active sites of CYPs (Chun et al., 2001; Regev-Shoshani et al., 2004).

Recently it has been shown by Wang et al., that resveratrol prevents acetaminophen-mediated liver damage by inhibiting CYP-mediated bioactivation of acetaminophen and regulating SIRT1, p53, cyclin D1, and PCNA to facilitate liver regeneration (Wang et al., 2015). Zhang et al., found that pretreatment of resveratrol effectively reversed As_2_O_3_-induced liver toxicity indices and resulted in a significant improvement in hepatic function in cat models. Moreover, resveratrol also improved the glutathione levels, activities of antioxidant enzymes and attenuated As_2_O_3_-induced increases in reactive oxygen species and malondialdehyde production in liver tissues (Zhang et al., 2014). Resveratrol has also been shown to offer protection against methotrexate, sodium fluoride and azozymethane- induced hepatotoxicity in animal models (Dalaklioglu et al., 2013; Gurocak et al., 2013; Atmaca et al., 2014).

## Curcumin

Curcumin is one of the most widely used herbal formulations to protect drug-induced toxicity. It is the major constituent of the spice turmeric extracted from the root of Curcumalonga Linn. It is metabolized to curcumin glucuronides, sulfates, tetrahydrocurcumin and hexacurcumin in the intestine and liver of human and rats and is reported to exhibit antioxidant, anti-inflammatory, choleretic, anti-carcinogenic, antiviral, and anti-infectious activities (Pan et al., 1999; Joe et al., 2004; Maheshwari et al., 2006). It inhibits the expression of the enzyme cyclooxygenase 2 via interference with activation of the transcription factor NF-κB and is a potential hepatoprotectant (Plummer et al., 1999; Duvoix et al., 2005; Aggarwal and Shishioda, 2006). Curcumin may be used in combination with conventional chemotherapeutic drugs to reverse multi drug resistance (MDR) in cancer cells and is the most effective MDR modulator among curcuminoids (Chearwae et al., 2004). Curcumin attenuates the proliferation of human lymphocytes and the production of several inflammatory mediators such as lipid mediators and cytokines and possess immunosuppressive and anti-rheumatic activity (Fahey et al., 2007; Gautam et al., 2007; Sandur et al., 2007). Curcuminoids have also been reported to inhibit pro-inflammatory induction by enhancing peroxisome proliferator-activated receptor-gamma (PPAR-gamma) activation (Jacob et al., 2007). Soliman et al., showed the curcumin-mediated protection against paracetamol-induced liver damage by restoring antioxidant activity of liver. They further showed that curcumin effectively restored the paracetamol-mediated increase in matrix metalloproteinase-8 (MMP-8), interleukin-1β (IL-1β), IL-8, tumor necrosis factor-α (TNF-α), and acute phase proteins and decrease in the expression of antioxidant genes (Soliman et al., 2014). Curcumin offers hepatoprotection against cisplatin, alcohol and heavy metals-induced liver damage in animals (Lee et al., 2013; García-Niño and Pedraza-Chaverrí, 2014; Waseem et al., 2014; Sankar et al., 2015; Soliman et al., 2015).

## Gingko

The Ginkgo biloba extract possess memory-enhancing, cognition-improving, immuno-modulating, apoptosis-inducing, and antiplatelet effects (Oken et al., 1998; Diamond et al., 2000; Wesnes et al., 2000; McKenna et al., 2001; Mahady, 2002; Andrieu et al., 2003; Ponto and Schultz, 2003; Yang et al., 2014a). The therapeutic benefits of this herbal medicine in neurological disorders, chronic refractory schizophrenia, hepatotoxicity, and in sleep disturbance of depressed patients have been reported (Hemmeter et al., 2001; Watanabe et al., 2001; De Smet, 2002; Yang et al., 2011). The major constituents of ginkgo include flavonoids (e.g., kaempferol), terpenoids (e.g., ginkgolides and bilobalaides), and organic acids (e.g., ginkgolic acids and alkylphenols) (Krieglstein et al., 1995; Jaggy and Koch, 1997; Tang et al., 2001; Lichtblau et al., 2002; Van Beek, 2002). As an adjuvant therapeutic drug, ginkgo appears to be promising in diabetics with respect to ischemic myocardium injury (Schneider et al., 2008). Intake of Ginkgo biloba extract may alter the hepatic metabolism by modulating hepatic drug metabolizing enzymes, altering the level of antioxidant enzymes and endogenous antioxidants such as GSH. The altered hepatic metabolism may result in the clearance of co-administered drugs, in particular, that have reduced renal and liver function (Deng et al., 2008).

## Other herbal medicines

Use of herbs in treatment of various liver disorders is common in China. The potential of these herbs against diseases with few and even no side effects continuously increases its popularity (Table 3). Now these medicines are being gradually accepted worldwide, particularly in Asia, Europe and North America. Nevertheless, the application strategy may differ in East and West due to variety of reasons, such as, philosophical viewpoint, concept of diseases, and treatment approaches.

**Table 3:** **A list of herbal medicines and their effects**.

**Herbal medicines**	**Effects**	**References**
Chai Hu Qing Gan Decoction	Ameliorates the central necrosis and fatty changes of the liver	Lin et al., 2007
Da Cheng Qi Decoction	Protects against gastrointestinal disorders, and liver diseases	Tseng et al., 2007
Xiao Cheng Qi Decoction	Protects against gastrointestinal disorders, and liver diseases	Tseng et al., 2007
Tiao Wei Cheng Qi Decoction	Protects against gastrointestinal disorders, and liver diseases	Tseng et al., 2007
Fenofibrate	Inhibits TNF-α and up-regulates PPAR-α and protects against NAFLD	Hong et al., 2007
Xuezhikang	Inhibits TNF-α and protects against NAFLD	Hong et al., 2007
Breviscapine extracted herb Erigeron breviscapus	Attenuating liver lipid accumulation and oxidative stress	Wu et al., 2007a,b
Inchinkoto	Increased hepatic levels of heme oxygenase-1 and GSH by a nuclear factor-E2-related factor (Nrf2)-dependent mechanism	Okada et al., 2007
Tanshinones	Decreases ALT and MDA levels, and increases ORAC, vitamin C and GSH levels in liver tissues	Xu et al., 2006
Root of Bupleurus spp.	Inhibitory capacity on superoxide anion formation and superoxide anion scavenging activity	Liu et al., 2006
BJ-JN (a traditional Chinese formulation)	ALT levels, hepatic NO and MDA content, and restores hepatic SOD activity and alleviates diminished (by toxin treatment) splenocyte proliferation	Zou et al., 2006
Piper betel leave	It has the biological capabilities of detoxication, anti-oxidation, and antimutation	Young et al., 2007
Cordyceps sinensis	Possesses the antitumor activity, antioxidant activity, and the capability of modulating the immune system	Chen et al., 2006
Baicalin	Decreases the leakages of LDH and ALT, and the formation of MDA; attenuates GSH depletion and oxidative stress. Histopathological evaluation of the rat livers revealed that baicalin reduced the incidence of liver lesions including hepatocyte swelling, leukocyte infiltration, and necrosis induced by toxin	Hwang et al., 2005
Crocetin	Protective action of crocetin operated via quenching of the superoxide anion and/or free radical	Tseng et al., 1995
*Bidens bipinnata* L.	Suppresses nitric oxide production and NF-κB activation, and possesses antioxidant property	Zhong et al., 2007
Mung bean, adzuki bean, black bean, and rice bean	Possess antioxidant property and suppresses LPO	Wu et al., 2001

In a review published in 2007 on Chinese medicine, the authors have summarized 274 species (in 216 genera among 92 families) of herbs possessing protective effect against liver toxicity (Wang et al., 2007). They further suggested a crude classification of these drugs in two groups; firstly, the main ingredients, for example silybin, osthole, cumarin, glycorrhizin, flavonoids and so on; and secondly, the supporting substances like sugars, amino acids, resins, tannins, and volatile oil.

## Liver injury associated with herbal products

The drug-induced liver injuries are debated since decades; the liver toxicity of certain herbal products has only recently been recognized. There are not many studies available on this alarming aspect of herbal products till date; however, the threat cannot be ignored as they may present with the same spectrum of liver pathologies as synthetic products (Stickel and Shouval, 2015). A large number of herbal and dietary products including Shou-Wu Pian (*Polygonum multiflorum*), *Breynia officinalis*, Germander (*Teucrium chamaedrys*), Chaparral (*Larrea tridentata*), *Actractylis gummifera*, Impila (*Callilepsis laureola*), Pennyroyal (*Mentha pulegium*), Greater celandine (*Chelidonium majus*), Kava (*Piper methysticum*), Black cohosh (*Cimifuga racemosa*), Noni juice (*Morinda citrifolia*), Gotu Kola (*Centella asiatica*), etc. are reported to have liver damaging effects (Stickel and Shouval, 2015). These products when consumed may cause symptoms ranging from acute, chronic, cholestatic, fulminant, and acute autoimmune-like hepatitis to acute liver failure, and liver cirrhosis. The mechanism of their toxicities are largely unknown; however, involvement of oxidative stress and apoptosis is also reported (Stickel and Shouval, 2015).

## Conclusion

The nature and dose of a particular drug are not the only determining factors of cell injury. Other factors such as an individual's gene expression profile, antioxidant status and the capacity for regeneration are also crucial. Several mechanisms are involved in the initiation of liver cell damage and aggravate ongoing injury processes. Dysfunction of these vital cell organelles results in impairment of dynamic equilibrium in homeostatic condition, thus resulting in intracellular oxidative stress with excessive formation of reactive oxygen species. Major causes of the hepatotoxic reactions by drugs are elevated ROS generation, oxidative stress and suppressed immune responses. Hepatotoxicity remains a major cause of drug withdrawal from the market. Recent examples in the USA and Europe are ximelagatran, nefazodone, nimesulide, ebrotidine, trovafloxacin, troglitazone, bromfenac, and so forth.

Natural products have shown great promise in combating against the toxicity of several commonly used drugs, including acetaminophen and paracetamol. Additionally, some of these natural products, such as resveratrol and curcumin, are now widely accepted chemopreventive agents. Due to easy availability and dietary nature, it is time to promote the natural products as supplementary medication with drugs that also cause toxicity to cells. Although a majority of natural products investigated to date are non-toxic, some studies have shown liver toxicity by certain natural products. Therefore, the proper selection of the natural products is also necessary. It is envisioned that natural products will not only lower the risk of drug-induced liver damage, but also provide an alternative solution to remedy the drug-induced hepatotoxicity.

## Author contributions

DS wrote the manuscript. WC and GU revised the manuscript.

### Conflict of interest statement

The authors declare that the research was conducted in the absence of any commercial or financial relationships that could be construed as a potential conflict of interest.
